# An Evaluation of the Impact of Psychiatry-Based High-Fidelity Simulation Training for Undergraduate Medical Students in the West Midlands

**DOI:** 10.1192/bjo.2022.141

**Published:** 2022-06-20

**Authors:** Sambavi Navaratnarajah, Helen Wheeldon, Amanda Brickstock, Emma Barrow

**Affiliations:** 1 Birmingham and Solihull Mental Health NHS Foundation Trust, Birmingham, United Kingdom; 2 University of Birmingham, Birmingham, United Kingdom

## Abstract

**Aims:**

Simulation (sim) is an excellent but underused tool suited to key skills in psychiatry such as communication, managing agitated patients and exploring the mental-physical health interface. Access to complex psychiatric patients has always been challenging and this has been exacerbated by the current COVID-19 pandemic. This has further increased fear amongst students creating another barrier to engaging with psychiatric patients. Our aim of the study was to evaluate the use of simulation within psychiatry as the literature in this field is underrepresented compared to other medical specialities. We hope to advocate its use in future undergraduate training.

**Methods:**

We developed 3 simulated scenarios for fourth year medical students; these involved identifying lithium toxicity and steroid-induced psychosis in ward settings and conducting an A&E risk assessment. The scenarios were developed following feedback from a focus group of foundation doctors on their psychiatry rotations. Data were collected pre- and post-simulation from a cohort of psychiatry students in this academic year. We assessed confidence levels in 7 domains using a 10-point Likert scale and obtained qualitative data to give context to the data collected.

**Results:**

81 and 83 students respectively completed the pre and post questionnaires. Quantitative data found that the student's confidence in all domains improved from pre to post simulation training. For example, confidence in performing a risk assessment improved from M = 4.12 to M = 7.04 and in making a basic management plan from M = 3.43 to M = 6.72. Qualitative data looked at skills gained, empathy and how the scenarios related to clinical practice. Key themes found improvements in de-escalation skills, handing over and self-reflection.

**Conclusion:**

The study supports the evidence that high-fidelity simulation is an important education tool in psychiatry. As facilitators, we feel that confidence scores improved due to the debrief. The standard tool often used is the diamond debrief however we found we had to adapt this model due to fourth year students not having developed sufficient skills to reflect on complex psychiatric scenarios. Therefore, an adjusted debrief was developed featuring technical knowledge and constructive feedback. In the future, we hope to explore the long-term benefits of simulation and its impact on clinical practice.

